# Sonic hedgehog stimulates neurite outgrowth in a mechanical stretch model of reactive-astrogliosis

**DOI:** 10.1038/srep21896

**Published:** 2016-02-23

**Authors:** Antonio Berretta, Emma K. Gowing, Christine L. Jasoni, Andrew N. Clarkson

**Affiliations:** 1 Department of Anatomy, Brain Health Research Centre, University of Otago, Dunedin 9054, New Zealand.; 2 Brain Research New Zealand, University of Otago, PO Box 913, Dunedin 9054, New Zealand; 3 Faculty of Pharmacy, The University of Sydney, Sydney, Australia

## Abstract

Although recovery following a stroke is limited, undamaged neurons under the right conditions can establish new connections and take on-board lost functions. Sonic hedgehog (Shh) signaling is integral for developmental axon growth, but its role after injury has not been fully examined. To investigate the effects of Shh on neuronal sprouting after injury, we used an *in vitro* model of glial scar, whereby cortical astrocytes were mechanically traumatized to mimic reactive astrogliosis observed after stroke. This mechanical trauma impaired neurite outgrowth from post-natal cortical neurons plated on top of reactive astrocytes. Addition of Shh to the media, however, resulted in a concentration-dependent increase in neurite outgrowth. This response was inhibited by cyclopamine and activated by oxysterol 20(S)-hydroxycholesterol, both of which modulate the activity of the Shh co-receptor Smoothened (Smo), demonstrating that Shh-mediated neurite outgrowth is Smo-dependent. In addition, neurite outgrowth was not associated with an increase in *Gli-1* transcription, but could be inhibited by PP2, a selective inhibitor of Src family kinases. These results demonstrate that neurons exposed to the neurite growth inhibitory environment associated with a glial scar can be stimulated by Shh, with signaling occurring through a non-canonical pathway, to overcome this suppression and stimulate neurite outgrowth.

Sonic Hedgehog (Shh) is a secreted glycoprotein, that has been shown to play a causal role in development through its ability to pattern the ventral central nervous system (CNS)^1^,^2^. In the canonical pathway, Shh binds to its receptor Patched (Ptc-1) to de-repress Smoothened (Smo) and activate transcription factors of the Gli family. During development Shh has been shown to play roles in cell proliferation, differentiation, migration, and cell survival^3^. Shh is also a key player in axon growth and guidance throughout the developing brain^2^,^4^,^5^. Shh appears to guide the pattern and number of axonal projections in a concentration-dependent manner using signaling pathways that are fundamentally different from the canonical Smo-Gli pathway. In this non-canonical pathway after binding to Ptc-1 and following de-repression of Smo, a transcription-independent cytoskeletal rearrangement occurs that stimulates the activation of src-family kinases^6^, to stimulate growth cone movement during axon development^4^. This direct action on nascent axons makes Shh a plausible candidate for facilitating axonal sprouting and recovery of function after injury such as stroke.

Following a stroke, surviving neurons within the peri-infarct region that surrounds the stroke core sprout new connections as part of the recovery process; however the mechanisms by which this happens have not been fully explored^7^,^8^,^9^,^10^. Stroke induces fully differentiated cortical neurons to switch into a growth mode: to sprout axons, elongate and form new connections^7^,^8^. This process may represent the re-activation of a developmental program, or it may involve unique adult regeneration-associated genes^9^. The ability of undamaged neurons to establish new connections after stroke is strongly inhibited by reactive astrocytes within the peri-infarct area^10^. After a trauma, reactive astrocytes express extracellular matrix proteins, like chondroitin sulfate proteoglycans (neurocan, versican and phosphacan), and developmentally-associated growth cone inhibitors (ephrins and semaphorins), which act as inhibitors of axonal regeneration^10^.

While the role of Shh is well documented during neurodevelopment, the effect of Shh in the adult or after injury (including stroke) is less well known. Recent evidence suggests that the Shh pathway is induced after injury^11^ and can facilitate changes in plasticity after stroke via an increase of tissue plasminogen activator, a protease that is considered responsible for the shh-mediated recovery^12^. To investigate the biological function of Shh signaling on neurite outgrowth after injury, we used an *in vitro* model where astrocytes were traumatized to render them reactive, similar to reactive astrogliosis found *in vivo* after stroke^13^,^14^. Postnatal cortical neurons were then cultured on top of reactive astrocytes in order to mimic their *in vivo* physical association. In particular, we used neurons collected from P5-6 mice that are more sensitive to the secreted glial inhibitors^13^,^14^ and evaluated whether Shh may be able to stimulate neurite outgrowth in this injury model.

## Results

### Shh increased neurite outgrowth of cortical neurons grown on reactive astrocytes

**Figure 1:**
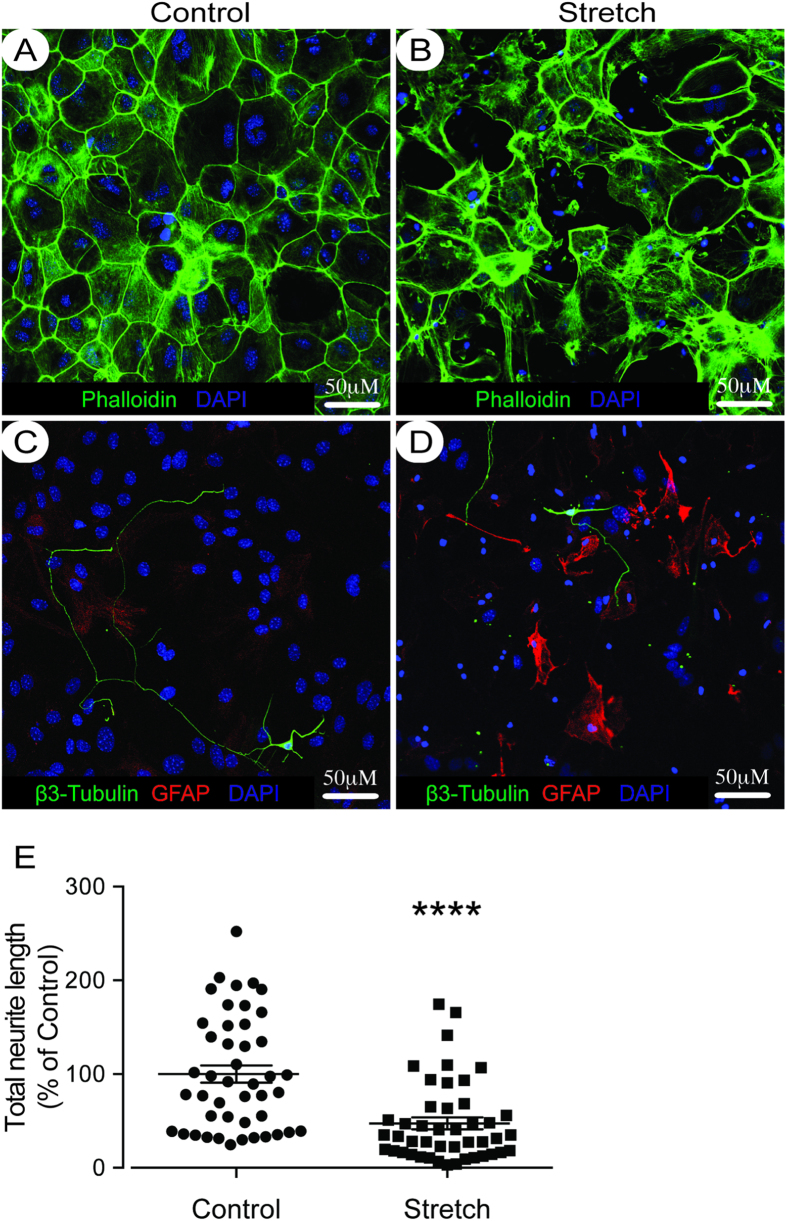
Mechanical trauma induces reactive astrogliosis and inhibits neurite outgrowth. Confocal images of the cytoskeleton marker F-Actin (labeled with Phalloidin, in green) and nuclear straining (in blue) for control astrocytes (**A**) and astrocytes 24-hours post-mechanical trauma (**B**) reveals extensive conformational changes in the stretched astrocytes. The effects of plating cortical neurons from P5-6 mice on top of either control (**C**) and stretched (**D**) astrocytes reveal impaired neurite outgrowth post-stretch. Reactive astrocytes are shown in red (GFAP), neurons in green (β3 tubulin) and the nuclear counter label in blue. Quantification of the total neurite lengths reveals impaired neurite outgrowth of neurons plated on top of stretched astrocytes (**E**). Data is expressed as mean ± SEM from 3 independent co-cultures, performed in triplicate (45 neurons per condition were analysed). ****P < 0.0001 versus control co-cultures.

The effect of activating the Shh-signaling pathway on neurite outgrowth was investigated using an *in vitro* model of reactive astrogliosis. This model consisted of mechanically traumatizing mature astrocytes cultured on deformable plates^13^,^14^, which resulted in morphological changes in the cytoskeleton (F-actin rearrangement; Fig. 1A,B) and up-regulation of GFAP expression (Fig. 1C,D).

To assess the effects that reactive astrogliosis had on cortical neuron outgrowth, post-natal neurons were plated at low-density 24-hours after rendering the astrocytes reactive. Neurites from these neurons were then quantified 24-hours later. Neurons plated on control (non-stretched) astrocytes were able to extend long neurites (Fig. 1C,E), whereas neurons that were plated on top of stretched astrocytes had reduced neurite outgrowth (Fig. 1D,E).

**Figure 2:**
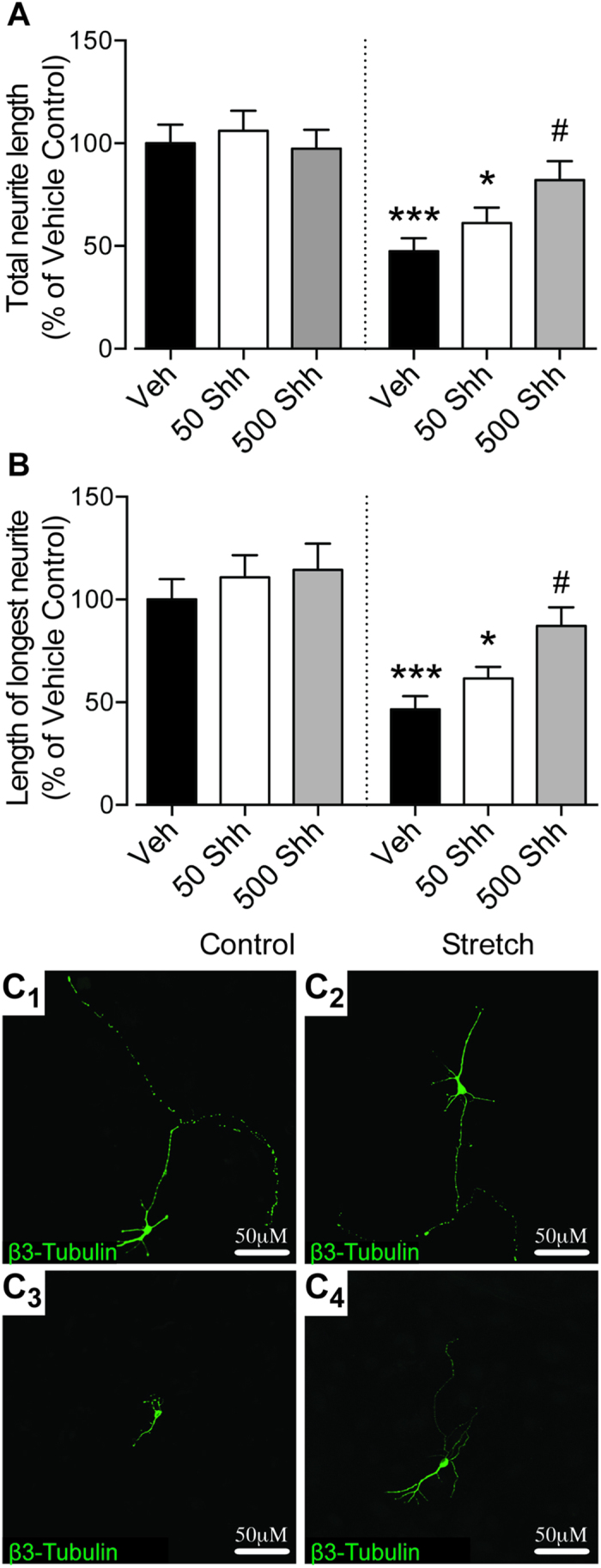
Shh increases neurite outgrowth of cortical neurons plated on top of stretched astrocytes. Quantification of total neurite lengths (**A**) and the length of the longest neurite (**B**), reveals that 24-hours treatment with 500 ng/mL Shh increases neurite outgrowth of neurons plated on top of stretched astrocytes but not control astrocytes. Representative confocal images of individual neurons are shown in Panel (**C**) for control + vehicle (**C1**), control + 500 ng/mL Shh (**C2**), stretch + vehicle (**C3**), and stretch + 500 ng/mL Shh (**C4**), scale bar represents 50μm. Data is expressed as mean ± SEM from 3 independent co-cultures, each performed in triplicate (45 neurons per condition were analysed). *P < 0.05, ***P < 0.001 compared with vehicle-treated control co-cultures; ^#^P < 0.05 compared with vehicle-treated stretch co-cultures.

We next investigated whether activation of Shh-signaling pathways that are involved in developmental neurite growth might be able to overcome this injury- and astrogliosis-induced neurite growth impairment. At 50 ng/mL, Shh did not significantly increase the total neurite length of neurons plated on either control or stretched astrocytes (Fig. 2A). However, administration of 500 ng/mL Shh to the media increased the total neurite length of neurons plated on stretched astrocytes when compared with vehicle-treated neurons plated on stretched astrocytes (Fig. 2A,C). The lengths of neurites of neurons plated on non-stretched astrocytes were unchanged by any concentration of Shh when compared with vehicle controls (Fig. 2A,C). In addition to total neurite length, the length of the longest neurite was also examined as this measures the growth of putative axons^15^. Similar to total neurite length, administration of 500 ng/mL Shh only resulted in a significant (nearly 50%) increase in the length of the putative axon of neurons plated in stretched astrocytes (Fig. 2B), and was not significantly different when compared to non-stretched control. The length of the putative axon of neurons grown on control astrocytes was unchanged in the presence of Shh (Fig. 2B).

### Shh-induced neurite outgrowth was mediated by a Gli-1 independent pathway engaged by Smo

**Figure 3:**
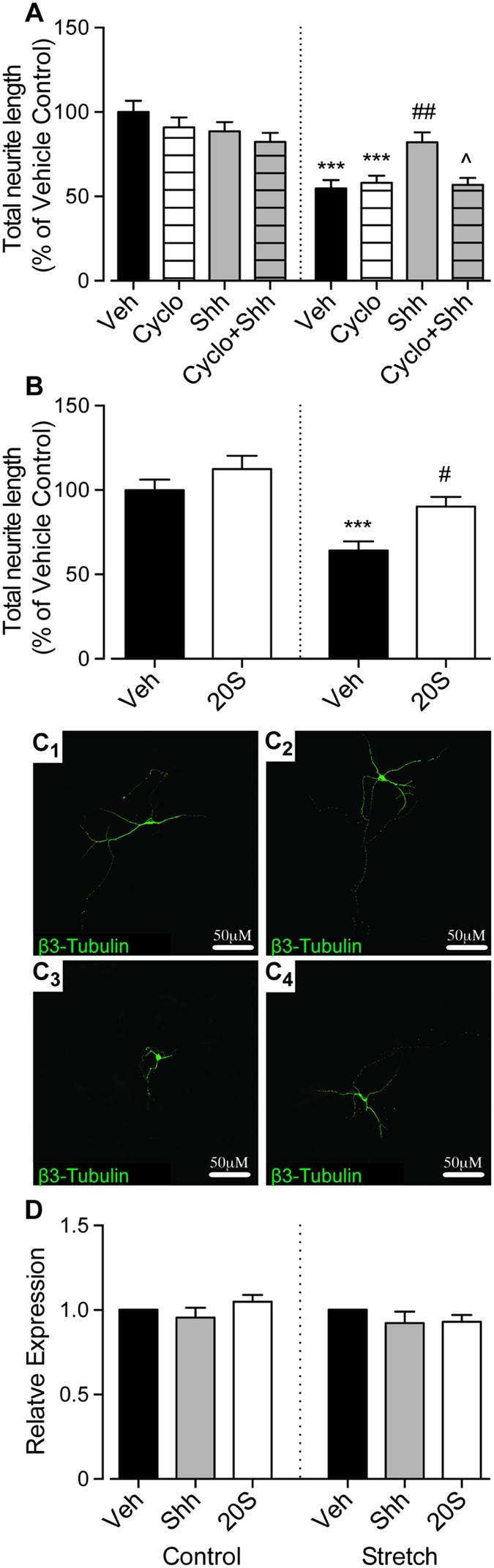
Shh-induced neurite outgrowth was mediated by Smo-dependent Gli-1-independent signaling. Quantification of the total neurite lengths showed that co-administration of 5 μM cyclopamine (antagonist of Smo) with 500 ng/mL Shh completely blocked the Shh-induced neurite outgrowth (**A**). Treatment with 20 S for 24-hours increased neurite outgrowth of cortical neurons plated on top of stretched astrocytes but not when they were plated on top of non-stretched control astrocytes (**B**). Representative confocal images of individual neurons are shown in Panel (**C)** for control + vehicle (**C1**), control + 5 μM 20 S (**C2**), stretch + vehicle (**C3**), and stretch + 5 μM 20 S (**C4**), scale bar represents 50μm. Assessment of *Gli-1* expression in non-stretched control and stretched co-cultures shows no change in expression following treatment with either 500 ng/mL Shh or 5 μM 20 S compared to vehicle-treated co-cultures (**D**). Data is expressed as mean ± SEM from 3 independent co-cultures, each performed in triplicate. For assessment of neurite length a total of 45 neurons per condition were analysed. ***P < 0.001 compared with vehicle-treated control co-cultures; ^#^P < 0.05 compared with vehicle-treated stretch co-cultures; ^^^P < 0.05 compared with Shh-treated stretch co-cultures.

To verify the role of Smo in astrocyte-neuron co-cultures responding to Shh we used two approaches. Firstly, cyclopamine (5 μM), a Smo inhibitor was co-administered with Shh, and was shown to block Shh-mediated neurite outgrowth (Fig. 3A). Cyclopamine was unable to modify neurite outgrowth significantly when applied alone to co-cultures (Fig. 3A).

Recent reports have suggested that sterol-like small molecules can act as allosteric modulators of Smo and can mimic Shh activity^16^. To further confirm the role of Smo in the neurite outgrowth response to Shh under conditions of reactive astrogliosis, we used the osteogenic oxysterol, 20(S)-hydroxycholesterol (20S)^16^,^17^,^18^. Similar to Shh, 5 μM 20 S increased neurite outgrowth of cortical neurons grown on reactive astrocytes, but not on control non-stretched astrocytes (Fig. 3B,C1–4).

Shh is known to signal through multiple pathways^4^,^6^,^19^. In the canonical pathway, Shh signaling causes a Smo-dependent increase of *Gli-1* gene expression, whereas in the non-canonical pathways Smo stimulates a *Gli-1*-independent response^19^. When control or stretched co-cultures were treated with either 500 ng/mL Shh or 5 μM 20 S for 24-hours, qPCR for *Gli-1* failed reveal any significant change of *Gli-1* expression (Fig. 3D). To verify that both 500 ng/mL Shh or 5 μM 20 S can induce *Gli-1* expression under the right conditions, 10T1/2 cells were cultured and subsequently treated with 500 ng/mL Shh or 5 μM 20 S, both of which were shown to induce *Gli-1* transcription (Supplementary Figure 1), as previously reported^18^.

Taken together, these observations establish that the Shh-dependent changes in neurite outgrowth observed here, were dependent on Smo, but independent of *Gli-1* transcriptional modulation, and suggest the involvement of a non-canonical Shh/Smo signaling pathway.

### Response of neurons to Smo activation requires non-canonical signaling through Src-family kinases

**Figure 4:**
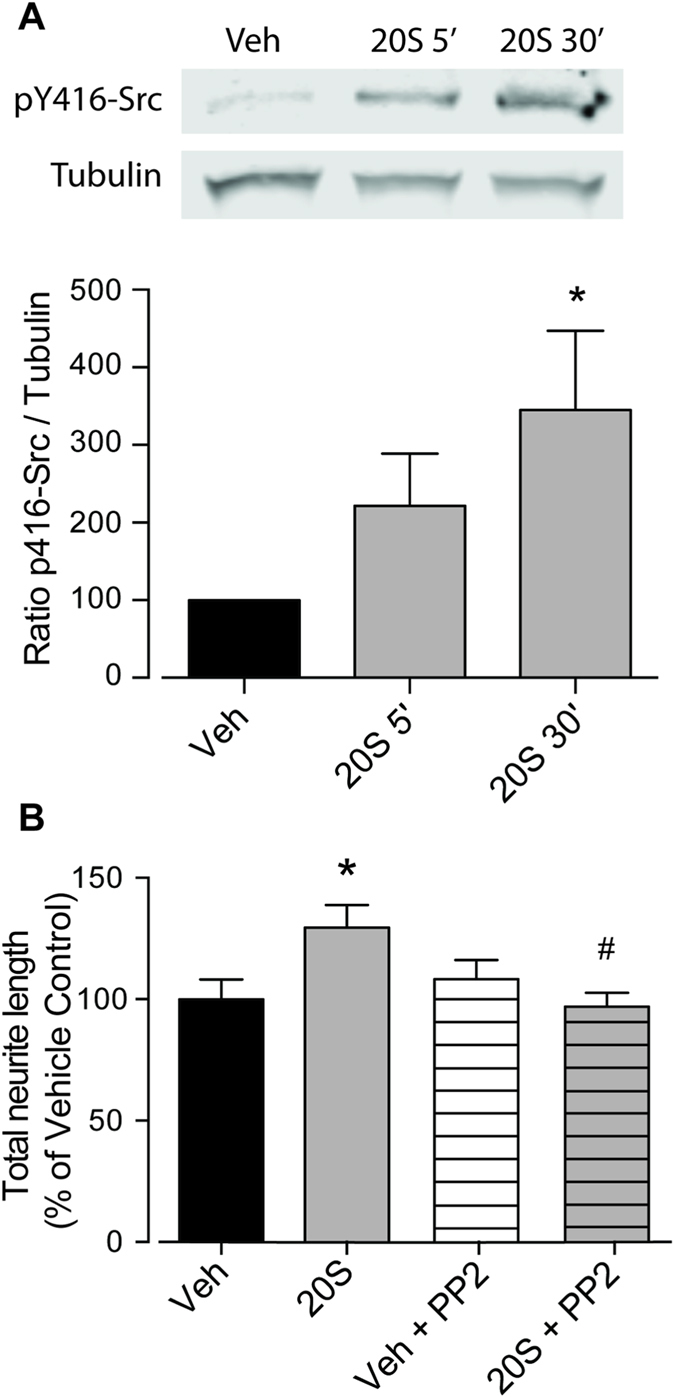
Smo-mediated increase of neurite outgrowth *requires non-canonical signaling through Src-family kinases.* Representative western blot showing a time-dependent increase in phosphorylation of Tyrosine 416 of Src (pY416-Src) in neuronal cultures after 5 and 30 min exposure to 5 μM 20 S. Alpha tubulin expression was used as loading control with densitometric ratios obtained to observe a difference from n = 4 independent experiments (**A**). Quantification of neurite outgrowth revealed that co-administration of 10 μM PP2 (an inhibitor of Src-family-kinases) with 5 μM 20 S to neurons plated on top of stretched astrocytes blocked the 20 S-mediated neurite outgrowth (**B**). Data for the assessment of neurite outgrowth was obtained from 3 independent co-cultures performed in triplicate (55 neurons per condition were analysed). ^*^P < 0.05 compared with vehicle-treated controls; ^#^P < 0.05 compared with 20 S-treated stretch co-cultures.

Recently, Smo-dependent, Gli-1 independent Shh-signaling pathways have been described^19^. Of particular interest is a non-canonical Shh-signaling pathway involving Src-family-kinases (SKFs) that is responsible for the control of axon growth and guidance during neurodevelopment^4^. Given these possibilities, we first confirmed the presence of the Smo-dependent Src-pathway in neurons by assessing the amount of Y416-Src phosphorylation, a previously described marker of SFK activity^20^, in pure neuronal cultures. Western blotting analysis showed that 30 min treatment with 20 S significantly increased Y416-Src phosphorylation (Fig. 4A), indicating that cortical neurons are able to respond to Smo activation. Neurons plated on post-trauma reactive astrocytes were then treated with 20 S either with or without the SFK inhibitor, PP2, which is known to inhibit the phosphorylation of Y416-Src (Supplementary Figure 2)^21^. Administration of 10 μM PP2 did not affect neurite outgrowth when applied alone to stretched co-cultures, but significantly reduced the neurite outgrowth stimulated by 20 S (Fig. 4B). This confirms that the Smo-dependent neurite outgrowth observed in conditions of reactive astrogliosis, is dependent on the activation of the non-canonical SFK-dependent pathway.

### Shh-induced neurite outgrowth was not mediated by changes in tPA levels or astrocyte apoptosis

**Figure 5:**
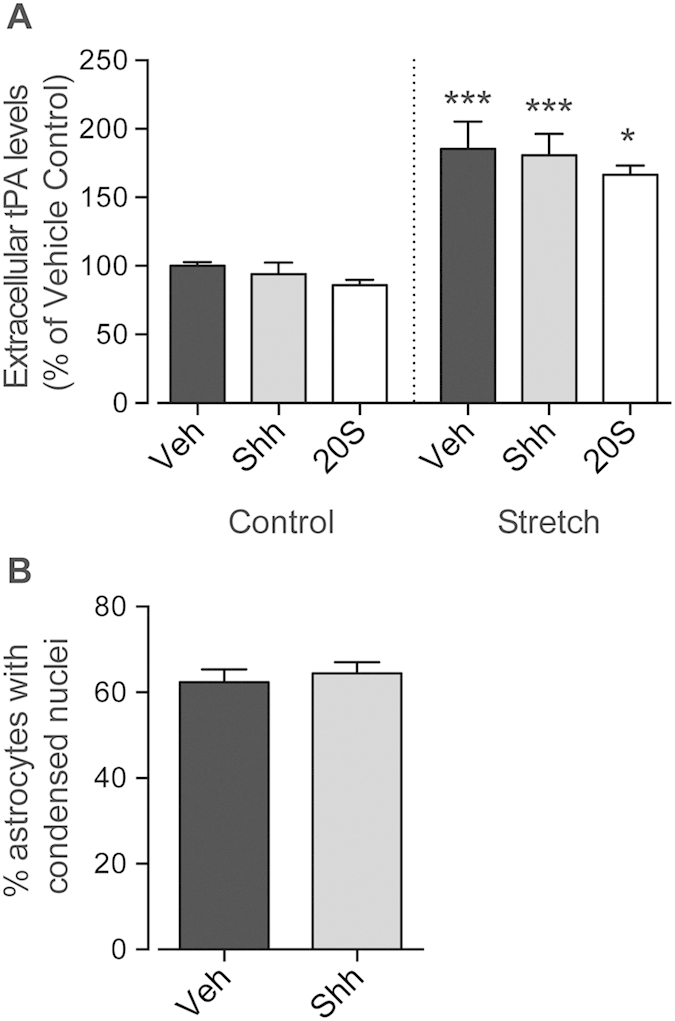
Shh-induced neurite outgrowth was not mediated by changes in extracellular tPA levels or astrocyte apoptosis. Assessment of extracellular tPA levels were shown to be elevated in the media from co-cultures where the astrocytes had been stretched compared with non-stretched astrocyte co-cultures. Treatment with either 500 ng/mL Shh or 5 μM 20 S failed to alter these levels (**A**). Quantifying the number of astrocytes with condensed nuclei was carried out as an assessment of astrocyte apoptosis. Treatment with 500 ng/mL Shh did not change the percentage of astrocytes showing condensed nuclei compared to vehicle-treated controls (**B**). Data obtained from 4 independent co-cultures. *P < 0.05, ***P < 0.001, compared to vehicle-treated control co-cultures.

Astrocytes express proteins that can positively or negatively modulate axonal sprouting. Since astrocytes express Ptch1 and Smo, we examined whether the effects of Shh on neurons could be occurring indirectly via a change in expression of other astrocytic modulators of neurite growth. tPA is reported to be released by astrocytes after Shh treatment and can increase neurite outgrowth^12^. To investigate the role of tPA in Shh-mediated neurite outgrowth we analyzed the levels of tPA in conditioned medium obtained from co-cultures. Analysis of the conditioned media from stretched astrocytes revealed a 70–100% increase in tPA levels compared to non-stretched controls (Fig. 5A). Treatment with either Shh or 20 S showed no differences in tPA levels 24 hours following treatment when compared to vehicle-treated stretched or non-stretched controls (Fig. 5A). Although tPA is increased when astrocytes are damaged, tPA levels were not altered in the presence of either Shh or 20 S, and therefore neurite growth in response to Smo-signaling is unlikely to be mediated by the release of tPA from astrocytes.

Apoptosis of astrocytes has previously been reported to contribute to reactive astrogliosis and influence neurite outgrowth^22^. In our stretch condition, 50–60% of the astrocytes were characterized as having nuclear condensation, an indication of apoptosis (Fig. 5B). Since the Shh-Smo pathway has been reported to reduce the apoptosis of cultured astrocytes^23^, we investigated whether the Shh-mediated neurite-outgrowth was due to decreased astrocyte apoptosis. Treatment with Shh did not change either the number or the percentage of astrocytes with condensed nuclei, suggesting that Shh-mediated increase of neurite outgrowth is not mediated by a modification in astrocyte apoptosis (Fig. 5B).

## Discussion

The processes associated with neurite outgrowth are highly influenced by changes in the cellular environment^24^. Astrocytes support neurons and facilitate neuronal maturation but when damaged, they can inhibit neurite outgrowth and impair recovery. Overcoming this astrocytic inhibition is a plausible therapeutic approach for improving recovery of brain function following injuries such as stroke^25^,^26^. In the present study we have reported that Shh increased both total neurite length and the length of the longest neurite from post-natal cortical neurons grown on top of reactive astrocytes. We observed that cyclopamine, an antagonist of Smo protein, blocked the Shh-mediated neurite sprouting demonstrating the involvement of Smo-activation. Further confirmation for the involvement of Smo was provided by the usage of 20 S, which is a member of the oxysterol family that allosterically activates the Smo protein^17^, and shown to also increase neurite outgrowth in stretch conditions. Using this co-culture model of glial scar^13^,^14^, we report further that the Smo-mediated increase in neurite outgrowth is occurring via a non-canonical pathway, as neurite outgrowth was independent of *Gli-1* transcription and could be blocked by the SFK inhibitor, PP2.

There is evidence for extensive cortical neuronal sprouting and re-mapping in sensory, motor, and pre-motor areas following both peripheral and central nerve injury in rodents and non-human primates^27^ and these changes are likely to be present in humans following stroke^28^,^29^. Stroke induces fully differentiated cortical neurons to switch into a growth mode: to sprout axons, elongate and form new patterns of connections^30^,^31^. This process may represent a re-activation of a developmental cellular program, or it may involve unique adult regeneration-associated genes^32^,^33^. The mechanisms of injury and repair after stroke are very different from other forms of CNS injury, and the relative roles of developmental- and regeneration-associated gene expression in axonal sprouting post-stroke is poorly understood. Here we focussed on the role of developmental program reactivation in post-stroke CNS repair, and did so by investigating the role of Shh/Smo-signaling, given its roles in developmental CNS patterning^1^,^5^, and axon growth and guidance^4^,^34^. We observed that application of Shh or 20 S to glial scar-impaired cortical neurons can promote the outgrowth of new neurites, which is consistent with the ability of the responding neurons to reactivate a developmental program in the context of post-stroke recovery. Similar to what we report here, targeting other developmental signaling pathways, such as Ephrin-A5, have also been shown to stimulate neurite outgrowth and facilitate post-stroke recovery^14^.

In further support of a role for Shh following brain injury, Shh expression has been shown to increase in adult hippocampal neural progenitor cells following stroke^35^. Reactive or injured astrocytes appear to be an important source of Shh^36^, and Shh expression is increased in cultured astrocytes after mechanical injury and in oxidative stress conditions^36^,^37^. Although Yang and colleagues report Shh levels reaching 500 pg/ml, this is only enough to synergistically de-differentiate astrocytes and is not enough to stimulate astrocyte rejuvenation^37^. Thus, activation of the Shh developmental program appears to be only one endogenous mechanism that promotes recovery following stroke. Interestingly, Shh may perform this role using both canonical and non-canonical signaling pathways, similarly to how it acts during development. For example, exogenous administration of Shh has been shown to stimulate neurogenesis, and to influence stem cell neural progenitor populations^38^,^39^, which is likely to occur via the canonical Shh-signaling pathway. By contrast, as we show here the effects of Shh on axonal sprouting appear to occur via the non-canonical pathway. Taken together these results clearly identify Shh/Smo-signaling as a key endogenous regulator of post-stroke recovery, and point to the importance of both canonical and non-canonical signaling pathways, recapitulating what occurs developmentally, in an effort to maximize the full regenerative effects of Shh post-stroke.

Despite the release of Shh following stroke, and the apparent ability of neurons and neural stem cells to respond to it, endogenous Shh-signaling alone does not seem to be powerful enough to overcome inhibitory factors that are also released following adult CNS injury^9^,^40^,^41^,^42^. Indeed, after an *in vitro* mechanical injury the increase in endogenous Shh levels are modest (approximately 500 pg/ml)^37^. This level of Shh is similar to that observed in our cell culture conditions (data not shown) and it is approximately 10-fold lower than the Shh concentration required to increase neurite outgrowth in our studies. Many of these inhibitory factors have been shown to be present in *in vitro* models of astrogliosis, and our *in vitro* results as well as those of others suggest that boosting the endogenous repair process by providing additional supportive factors, such as Shh, is a tenable strategy for repair. This is consistent with reports showing that bone marrow-derived stromal cell transplants, which increase Shh, are able to facilitate post-stroke recovery^12^,^43^. In these studies, however, the effects of Shh on neurons appeared to be indirect, through stimulation of tPA release and astrocyte remodeling. By contrast, although we observed Shh/Smo-dependent neurite growth following injury, we did not observe a increase in tPA release in our cultures. This likely explains why we were able to observe the novel involvement of the non-canonical signaling pathway. Together these results indicate that Shh activates multiple pathways to play a multi-pronged role in repair after brain damage; however it is present in insufficient quantities endogenously to drive substantial recovery. Thus, our results point to the activation of Smo as a method to enhance Shh/Smo activity, and thereby offer a potential strategy for facilitating the post-trauma increase of axonal sprouting via the activation of non-canonical Shh-signaling. To assess the translatable potential of Shh, further studies are required to assess whether administration of Shh stimulates neurite outgrowth in *in vivo* models of injury via canonical or non-canonical pathways.

## Material and Methods

### Animals

All procedures described in this study were approved by the University of Otago, Animal Ethics Committee in accordance with the guidelines on Care and Use of Laboratory Animals (NIH Publication No. 85–23, 1996), and in accordance with the ARRIVE guidelines. Mice (C57BL/6J) were housed under a 12-hour light, 12-hour dark cycle (lights on 0600 h) with *ad libitum* access to food and water. Female mice were time-mated with age-matched males, and the day of vaginal plug detection was considered gestational day 0.5.

### Cortical astrocyte cultures and stretch trauma model

Astrocyte stretch trauma was performed as previously reported with minor modifications^13^,^14^. Astrocyte cultures were prepared from cortices of postnatal day (P)1–2 mice. The cortices were isolated, incubated for 10 min at 37 °C with 0.25% trypsin (Life Technologies Carlsbad, California, United States). Trypsin was inactivated with DMEM/F12 (Life Technologies) + 10% fetal bovine serum (FBS, New Zealand origin; Life Technologies). Cells were then dissociated by gentle pipetting in DMEM/F12, supplemented with 1% Penicillin/Streptomicin (Life Technologies) and 10% FBS, and plated (2 cortices per flask) in 75 mm^2^ flasks (Corning). The medium was changed every 3–4 days. After 10–12 days, cultures were shaken for 24–36 hours (180 rpm) and treated with 10 mM leucin methyl ester (Sigma-Aldrich, Castle Hill, Australia) for 12-hours to remove oligodendrocyte precursors and microglia. Secondary astrocyte cultures were then established by trypsinizing the primary cultures, and subplating 300,000 cells onto 35 mm-diameter deformable membrane wells coated with collagen I (Bioflex 6 well plates, Flexcell International, McKeesport, PA, USA). After 1-week the percentage of FBS in the medium was reduced to 5%, and after an additional 1–2 weeks the serum was reduced to 0.5% for 48-hours and then the cells were grown in the absence of serum. Six-hours after the removal of serum, the cells were mechanically traumatized using an abrupt pressure pulse with a pneumatic device (Flexcell FX-4000 Strain Unit, Flexcell International, McKeesport, PA, USA) programmed to produce a maximal elongation of 23% (130 ms, triangular stretch).

### Post-natal cortical neuronal cultures

Twenty-four hours after the mechanical trauma, cortical neurons were isolated from P5-6 mice and plated on top of the reactive astrocytes as previously reported^13^,^14^. Cortices were dissected in Hibernate medium (Life Technologies) and incubated for 30 min at 30 °C with 2 mg/ml papain (Sigma-Aldrich, Australia). After enzymatic digestion, cells were dissociated by gentle pipetting and the cellular suspension was purified by Optiprep (Sigma-Aldrich) gradient to isolate neurons from the other cells as previously reported^13^,^14^. Cortical neurons thus dissociated were plated at a density of 20,000/well on the top of pre-prepared astrocyte cultures.

### Pharmacological treatments

All pharmacological treatments were performed for 24-hours in medium containing 0% FBS. Recombinant mouse Shh, N-terminal peptide (R&D Systems Inc, Minneapolis, MN, USA) was resuspended in PBS and used at a final concentration of 50–500 ng/mL. 20(S)-Hydroxycholesterol (Tocris, Bristol, UK) and cyclopamine (Sigma-Aldrich) were reconstituted in ethanol and used at 5 μM. PP2 (Tocris) was resuspended in DMSO and used at 10 μM. Ethanol and DMSO were used at final concentration no higher than 0.1% and 0.04%, respectively.

### Immunocytochemistry

Cultures were fixed with 4% paraformaldehyde (PFA) for 10 min at room temperature (RT). Cultures were incubated with phosphate buffered saline (PBS) containing 0.2% Triton X-100 (Sigma-Aldrich) for 10 min at RT and then with blocking solution (PBS containing 2% bovine serum albumin (Sigma-Aldrich)) for 30 min at RT with gentle shaking. Subsequently, cultures were incubated for 2–3 hours a RT with mouse anti-β3 tubulin (TuJ1 clone, 1:2000, R&D Systems, Inc., Minneapolis, USA) and chicken anti-GFAP (1:5000, Millipore) antibodies. After washing (3 × 10 min in PBS at RT) cultures were incubated for 2-hours at RT with 488 and 549 DyLight dyes conjugated to affinity-purified secondary antibodies (1:1000, Jackson ImmunoResearch). Hoechst 33258 (0.4 μg/ml, Sigma-Aldrich) was used for nuclear counterstaining. For F-actin staining, cultures were incubated for 2–3 hours a RT with 488 Fluorescent Phalloidin (1:500 in PBS; Cytoskeleton, Inc).

### Neurite outgrowth Assay

For each well, all neurons (around 15–30 TuJ1-positive cells) in a randomly selected 110 mm^2^area were photographed digitally using an inverted fluorescence microscope with a 20x objective (IX71 Olympus, Tokyo, Japan). Neurite outgrowth was quantified using ImageJ (1.45 S version, NIH). The total neurite and the longest neurite lengths were measured. The measurements of the 5 neurons with the longest neurites per well were selected and analyzed. The analysis was performed by analyzing at least 45 neurons per condition across three independent co-culture preparations performed in triplicate.

### ELISA detection of active tPA

The level of functionally active tissue plasminogen activator (tPA) was determined using a mouse tPA activity assay kit (Innovative research, United States). Conditioned media was collected from neuron-astrocyte co-cultures 24-hours post-plating cortical neurons, and analyzed based on the manufacturer’s guidelines.

### RNA extraction and quantitative real-time PCR

*Gli-1* mRNA levels were assessed by quantitative real-time polymerase chain reaction (qPCR). Total RNA from neuron-astrocyte co-cultures was extracted using the Qiagen RNeasy kit and genomic DNA was removed using DNAse (Ambion) following the manufacturer’s protocols. The purity (RNA with ratio of absorbance at 260 nm and 280 nm ≥ 2) and amount of the RNA was measured spectrophotometrically (NanoDrop 2000, Thermo Scientific, USA). Total RNA (750 ng) was used to synthesize the first strand complementary DNA (cDNA) using Super Script III (Life Technologies) following the manufacturer’s protocol. After reverse transcription, the cDNA was amplified by qPCR using SyBr green master mix (Applied Biosystems, Foster City, CA) and the following primers (250 nM): *Gli-1*: for: TCC ACA CGC CCC CTA GTG rev: TGG CAA CAT TTT CGG TGA TG; *Gapdh*: for: GTC TAC TGG TGT CTT CAC CAC CAT rev: GTT GTC ATA TTT CTC GTG GTT CAC. qPCR was performed on a Roche Lightcycler (Roche, Minneapolis, MN) with the following cycling parameters: 40 cycles of 95 °C, 15 s; 60 °C, 30 s; 72 °C, 40 s. After amplification, a denaturing curve was performed to ensure the presence of unique amplification products. All reactions were performed in triplicate. Expression of mRNA was assessed by evaluating threshold cycle (CT) values. The CT values were normalized with the expression level of GAPDH, and the relative amount of mRNA specific to each of the target genes was calculated using the Δ−CT method^44^.

### Culture of pure cortical neurons from embryos

Cultures of pure cortical neurons were obtained from mice at embryonic day 14.5. In brief, cortices were isolated and treated with 0.125% trypsin + EDTA (Life Technologies) for 5 min at 37 °C. Trypsin was inactivated with DMEM/F12 + 10% FBS. Cells were then dissociated by gentle pipetting in Neurobasal medium (Life Technologies) supplemented with 0.5% Glutamax (Life Technologies), 1% Penicillin/Streptomicin, and 2% B27 supplement, and 600,000 cells/well were plated in 35 mm dishes (Nalge Nunc International, Rochester, NY) precoated with 0.1% gelatin (Sigma-Aldrich) and 50 μg/ml poly-L-lysine (Sigma-Aldrich). After 5-days *in vitro*, neurons were treated with 5 μM 20 S for 5 or 30 min. 10T1/2 cells were cultured and used as positive controls for *Gli-1* expression (see supplementary methods).

### Western blotting

Neurons were lysed with ice-cold RIPA buffer (Sigma-Aldrich) supplemented with protease (Complete, Roche; 1 pill per 10 ml of lysis buffer) and phosphatase inhibitors (Phostop, Roche; 1 pill per 10 ml of lysis buffer). Lysates were centrifuged at 14,000 *g* for 10 min at 4 °C and supernatants were stored at −80 °C. Total protein concentration was determined by DC Kit (Bio-Rad Laboratories, Inc.) and 10 μg of total proteins were mixed with a same volume of sample buffer and incubated at 95 °C for 5 min and loaded into 10% PAGE gels (Mini-protean TGX precast gels, Bio-Rad Laboratories, Inc.). Proteins were transferred to PVDF membrane using Trans-Blot Turbo Transfer System (Bio-Rad Laboratories, Inc.). Blots were blocked for 30 min using Odyssey Blocking Buffer (LI-COR Biosciences, Lincoln, NE, USA) and incubated with primary antibody for 2-hours at room temperature. The primary antibodies used were: rabbit anti-phospho Src-pY418 (Life Technologies, 1:1,000) and mouse anti-alpha tubulin (Abcam, 1:10,000). IRDye infrared secondary antibodies (LI-COR Biosciences) were used at concentration 1:10,000–1:20,000. Immunoreactivity was captured and quantified using the Odyssey Infrared imaging system (LI-COR Biosciences).

### Statistical Analysis

Statistical significance was assessed by one-way analysis of variance (ANOVA) followed by the Tukey post-hoc test (GraphPad Software, San Diego, CA) or by Student’s t-test when only two groups were compared. Data shown in the figures are the results of at least three independent experiments. Data are represented as mean ± SEM. P < 0.05 was considered significant for all analyses.

## Additional Information

**How to cite this article**: Berretta, A. *et al.* Sonic hedgehog stimulates neurite outgrowth in a mechanical stretch model of reactive-astrogliosis. *Sci. Rep.* **6**, 21896; doi: 10.1038/srep21896 (2016).

## Supplementary Material

Supplementary Information
