# Supplementary material for: Sonic hedgehog stimulates neurite outgrowth in a mechanical stretch model of reactive-astrogliosis
Source: Sci Rep. 2016 Feb 23;6:21896. doi: 10.1038/srep21896 (PMC4763245; doi:10.1038/srep21896)
Supplement: Supplementary Information [file srep21896-s1.pdf]

Sonic hedgehog stimulates neurite outgrowth in a mechanical stretch model of reactive-astrogliosis

Antonio Berretta, Emma K. Gowing, Christine L. Jasoni, Andrew N. Clarkson.

Supplementary Methods:

10T1/2 cells:

Clonal mouse embryo fibroblasts, C3H/10T1/2 (clone 8, American Type Culture Collection, ATCC, USA) were cultured as previously reported with minor modifications [1]. Cells were plated in six-well plates with initial plating density of 300,000 cells/well and grown in DMEM/F-12 medium containing 10% fetal bovine serum and penicillin-streptomycin. For differentiation, cells were induced 0.5mM IBMX and G5 supplement (1% concentration, Life Technologies).

Supplementary Results:

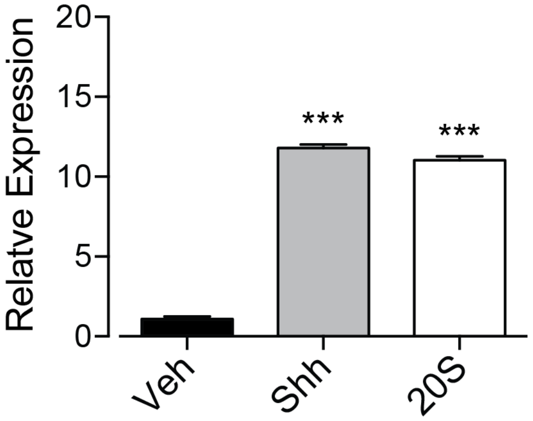

Supplementary Figure 1. Shh and 20S increased expression of *Gli-1* in 10T1/2 cells. *Gli-1* expression was assessed 24-hours following treatment of 10T1/2 cells. Addition of either 500ng/mL Shh or 5µM 20S to the media resulted in a significant increase in expression of *Gli-1* compared to vehicle treated control cells. *** $P<0.001$ compared to vehicle treatment.

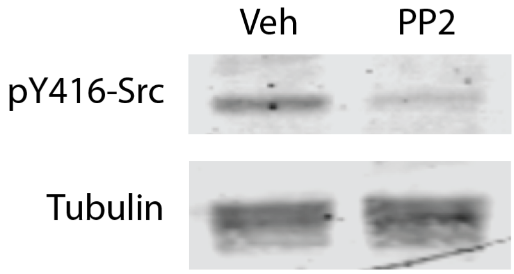

Supplementary Figure 2. PP2 reduces the phoshorylation of Y416-Src in 10T1/2 cells. To confirm that PP2, which is an inhibitor of SKF activity, was working, we tested the effects of PP2 on phospho-Y416-Src levels in differentiated 10T1/2 cells. Assessment of protein levels for phospho-Y416-Src shows a decrease in activity following 24-hours of 10µM PP2 treatment.

References

1 Liu, X. & Jefcoate, C. 2,3,7,8-Tetrachlorodibenzo-p-dioxin and Epidermal Growth Factor Cooperatively Suppress Peroxisome Proliferator-Activated Receptor-γ1 Stimulation and Restore Focal Adhesion Complexes during Adipogenesis: Selective Contributions of Src, Rho, and Erk Distinguish These Overlapping Processes in C3H10T1/2 Cells. *Mol Pharmacol* **70**, 1902-1915, (2006).
